# Automatic extraction of mesenchymal stromal cells from single-cell RNA-sequencing data of human dental pulp

**DOI:** 10.1186/s13040-026-00581-x

**Published:** 2026-07-07

**Authors:** Daigo Okada, Tomoko Takeda-Kawaguchi, Ken-Ichi Tezuka

**Affiliations:** 1 Stem Cell and Regenerative Medicine, Gifu University Graduate School of Medicine, Yanagido 1-1, Gifu, Japan; 2 Oral and Maxillofacial Surgery, Gifu University Graduate School of Medicine, Gifu, Japan; 3 Center for One Medicine Innovative Translational (COMIT) Research, Gifu University Institute for Advanced Study, Gifu, Japan; 4 Division of Preemptive Food Research, Preemptive Food Research Center (PFRC), Gifu University Institute for Advanced Study, Gifu, Japan; 5 National Institute of Informatics, Tokyo, Japan

**Keywords:** Single-cell RNA-sequencing, Dental pulp, Mesenchymal stromal cells

## Abstract

**Background:**

Human dental pulp is a complex tissue composed of diverse cell types, including mesenchymal stromal cells (MSCs), which are crucial for tissue repair and regeneration. Although single-cell RNA sequencing (scRNA-seq) data from human dental pulp have accumulated in recent years, MSC identification relies on manual verification of marker genes after clustering, which limits analytical efficiency and scalability. The choice of clustering resolution is determined empirically, potentially leading to under- or over-segmentation of MSCs or their mixing with other cell types. The objective of this study was to establish a computational workflow that automatically extracted uncultured MSCs from human dental pulp scRNA-seq data and to investigate the characteristics of freshly-isolated MSCs in dental pulp using multiple public datasets.

**Results:**

A computational workflow was developed that automatically identified MSC populations given a predefined marker set from a scRNA-seq count matrix and systematically evaluated the performance of the marker set. The MSC marker set consisting of six genes (*NT5E*, *THY1*, *ENG*, *FRZB*, *NOTCH3*, *MCAM*) demonstrated higher cluster separation than the three basic MSC markers (*NT5E*, *THY1*, *ENG*) and consistently detected an MSC population across multiple independent dental pulp scRNA-seq datasets. Pseudo-bulk transcriptomic analysis of MSC populations extracted using this workflow indicated that MSCs in the dental pulp of patients with pulpitis themselves exhibited an inflammatory phenotype, characterized by substantial activation of inflammation-related pathways. Donor aging was associated primarily with reduced mesenchymal identity and metabolic alterations.

**Conclusions:**

This study provides a framework for the automated extraction of dental pulp-derived MSCs and cross-dataset analysis. This framework is broadly applicable to the automated extraction of cell populations, such as dental pulp–derived MSCs, for which the annotation method is not yet well established, and is expected to be widely useful in single-cell analyses.

**Supplementary Information:**

The online version contains supplementary material available at 10.1186/s13040-026-00581-x.

## Background

Human dental pulp is a complex tissue composed of diverse cell types, including immune, endothelial, and nerve cells, fibroblasts, and mesenchymal stromal cells (MSCs), which contribute to tissue repair and regeneration [1]. MSCs are present in various tissues, such as bone marrow, adipose tissue, the umbilical cord, and dental pulp, and have been widely investigated for cell therapy applications due to their self-renewal capacity, multipotency, and immunomodulatory properties [2]. Dental pulp–derived MSCs can be relatively easily obtained from extracted teeth that are clinically discarded and have attracted attention as a promising cell source for regenerative medicine [3–5]. Mesenchymal stromal cells (MSCs) isolated from the dental pulp and expanded in culture have been widely investigated for their therapeutic potential in a broad range of conditions, including bone fractures, spinal cord injury, and periodontitis [6, 7]. Although MSCs are typically expanded in vitro prior to therapeutic use, culture procedures can alter their transcriptional states and functional properties [8, 9]. To better understand the intrinsic characteristics of dental pulp MSCs, it is important to investigate not only cultured MSCs but also freshly-isolated MSCs in the dental pulp that more closely reflect their in vivo state.

In recent years, the single-cell RNA sequencing (scRNA-seq) studies on human teeth have been actively reported [10, 11], providing valuable resources for the analysis of freshly-isolated cells in dental pulp. These studies have indicated the cellular heterogeneity of dental pulp at single-cell resolution, including its stromal, immune, vascular, and neural components. Previous scRNA-seq analyses of human dental pulp have described the overall cellular landscape [12–15], examined transcriptional changes associated with aging [16] and development [17], and investigated disease-related alterations, such as pulpitis [18]. Although these studies provide an important foundation for understanding the biology of human dental pulp, studies focusing on the systematic identification and characterization of MSCs in human dental pulp are limited.

When using such datasets to obtain information on freshly-isolated MSCs, it is necessary to first identify the MSC population. In dental pulp scRNA-seq datasets, MSCs are typically identified as distinct clusters following unsupervised clustering. Although cell-type annotation is often conducted manually by referencing known marker genes, such post hoc procedures can be time-consuming and subject to variability [19–21]. Several automated annotation methods, such as SingleR, Azimuth, and CellTypist, have been developed and are widely used [22–24]. While these approaches are highly effective when appropriate reference atlases are available, their performance depends fundamentally on the availability and relevance of such references. In the case of dental pulp, particularly dental pulp–derived MSCs, suitable reference datasets are scarce because current atlases do not include dental pulp–specific annotations, and many models are trained primarily on well-characterized tissues, such as blood or immune systems.

Consequently, analyses of dental pulp scRNA-seq datasets commonly rely on a workflow in which clusters are first identified by clustering and subsequently annotated based on marker gene expression. Although this approach is practical, it introduces an additional layer of uncertainty, the identification of MSCs depends not only on the choice of marker genes but also on the clustering resolution. Overly-coarse clustering may obscure MSC populations by mixing them with other cell types, whereas overly-fine clustering may fragment MSCs into multiple subclusters. Therefore, an automated strategy that enables robust and reproducible extraction of MSC populations while accounting for clustering granularity would facilitate the systematic analysis of dental pulp scRNA-seq data.

In this study, an MSC marker set and computational workflow were established to automatically extract high-purity, freshly-isolated MSCs from dental pulp scRNA-seq data and these were applied to multiple public datasets. By calculating the MSC marker module scores and optimizing clustering based on the separation between the highest and second-highest scoring clusters, a single MSC-annotated cluster was reproducibly identified. Pseudo-bulk analyses of the extracted MSC populations enabled the assessment of transcriptomic features associated with pulpitis and donor aging.

## Methods

### Preprocessing of single cell RNA-seq data

The raw scRNA-seq count data were analyzed using Seurat (version 5.0.1) [25]. Cells with nFeature_RNA * > * 200 and percent.mt * < * 10 were used for downstream analysis. The data were normalized using the NormalizeData function with a scale factor of 10,000. Highly variable genes were identified using the FindVariableFeatures function with the vst method, and the top 2,000 genes were selected. Principal component analysis (PCA) was conducted and the results were visualized using uniform manifold approximation and projection (UMAP) [26].

### Automatic detection workflow for MSCs from human scRNA-seq data

**Fig. 1:**
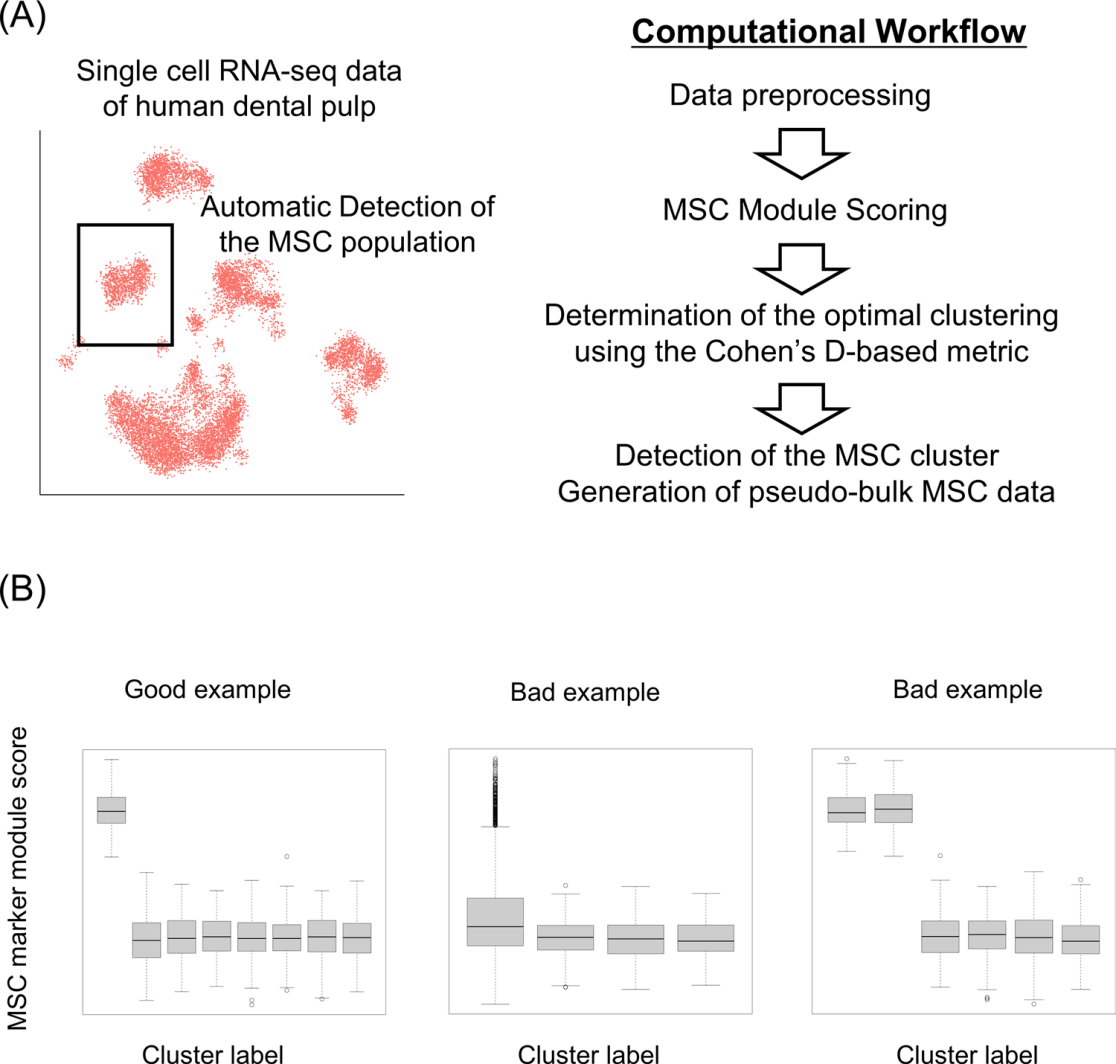
Conceptual schematic illustrating the research purpose and analytical concept. (**A**) Overview of the analytical workflow and its objectives. (**B**) Hypothetical examples of box-and-whisker plots of module scores by cluster under good vs. poor clustering conditions. Optimal clustering is defined as the configuration in which MSC marker gene module scores are selectively elevated in a single cluster

An automatic detection workflow for MSCs was developed from the count data of human scRNA-seq data from dental pulp (Fig. 1A). The top 50 principal components from the PCA were used for subsequent analyses. The module scores for the specified MSC marker set were calculated using the AddModuleScore function in Seurat.

To identify MSC clusters automatically, the number of clusters was optimized using a biologically-motivated criterion. Ideally, a single cluster should exhibit a high MSC marker module score, whereas all other clusters should remain uniformly low (Fig. 1B). Such a profile indicates a clear separation between MSC and non-MSC populations, enabling clear annotation of the high-scoring cluster as an MSC population. If the clustering resolution is too low, MSC and non-MSC cells are mixed within the MSC cluster, resulting in a flattened module score profile. Conversely, if the resolution is too high, the MSC population is fragmented into multiple clusters, which complicates the annotation. To quantitatively evaluate the clustering quality, we defined an optimized score as Cohen’s d between the clusters with the highest and second-highest average MSC module scores. Maximizing this score selects the optimal number of clusters that best isolates a single well-separated MSC cluster. This framework is applicable to any predefined cell-type marker gene set and was designed as a general approach for extracting a target cell population with high purity.

Based on this concept, an automated MSC detection procedure was implemented. K-means clustering was conducted on the principal component score matrix for *K = 5*–20. Clustering was conducted using the kmeans function with parameters nstart = 100 and iter.max = 200. Average module scores were calculated for each cluster. Cohen’s *d* between clusters with the highest and second-highest average module scores was calculated using the R package, effsize (version 0.8.1). The clustering result corresponding to the *K* value that maximized the optimized score was selected. When applying graph-based clustering, we employed the Louvain algorithm implemented in Seurat via the FindClusters function. The resolution parameter was systematically explored from 0.1 to 1.0 in increments of 0.1, and the optimal resolution was determined based on the predefined optimization criterion. Under the selected number of clusters or resolution parameter, the cluster with the highest average module score was identified as an MSC cluster. For this MSC cluster, the raw count values of all genes across cells within the cluster were pooled to generate pseudo-bulk count values representing the MSC population in the dental pulp sample.

### MSC identification using reference-based annotation tools

For comparison with the proposed workflow, MSC identification was additionally performed using a reference-based automated annotation tool. To evaluate a representative reference-based annotation approach, SingleR (version 2.4.0) was employed together with the celldex package (version 1.12.0) [22].

### Differential gene expression analysis for pseudo-bulk RNA-seq data

A differential gene expression analysis was conducted on the MSC pseudo-bulk RNA count matrix extracted using the proposed workflow. Genes with a total count *≤ 10* across all samples were excluded from the analysis. Fold change and statistical significance were calculated for each gene using the DESeq2 package (version 1.42.1) [27].

### Gene set enrichment analysis

Gene set enrichment analysis (GSEA) was conducted using the clusterProfiler package (version 4.10.0) with 10,000 permutations [28]. Hallmark gene sets were used as the reference database [29]. Gene sets with adjusted *P < 0.05* were considered statistically significant.

## Results

### A workflow for automatic extraction of MSCs from dental pulp scRNA-seq data

We used two public dental pulp scRNA-seq datasets: GSE202476 (13-year-old donor) [13] and GSE224676 (27-year-old donor) [14] to test our approach. These datasets were selected to evaluate whether the proposed workflow could reproducibly identify MSC populations across independent human dental pulp scRNA-seq studies. We evaluated a representative reference-based annotation method (SingleR) on these datasets (Supplementary Fig. 1). In GSE202476, MSC annotations were distributed across multiple regions in the UMAP space, and in GSE224676, MSCs were not detected. These observations suggest that reference-based annotation may not consistently capture MSC populations in dental pulp scRNA-seq data.

We applied the proposed automatic extraction workflow to datasets using two MSC marker sets (details are provided in the Methods section). The first consists of three canonical MSC markers, *ENG* (CD105), *THY1* (CD90), and *NT5E* (CD73), which constitute the minimal criteria defined by the ISCT [30]. The second was an expanded six-gene marker set constructed in a stepwise manner based on prior biological knowledge, consisting of these three canonical markers plus *FRZB*, *NOTCH3*, and *MCAM*, all of which have been reported to be MSC-associated markers in the dental pulp [15, 31, 32]. Notably, the selection of marker sets in this study was guided by prior knowledge and practical considerations, and should be regarded as a heuristic choice rather than a definitive or universally optimal definition of MSC identity. This expanded set was further evaluated within the proposed framework to assess the contribution of markers beyond the canonical definition.

**Fig. 2:**
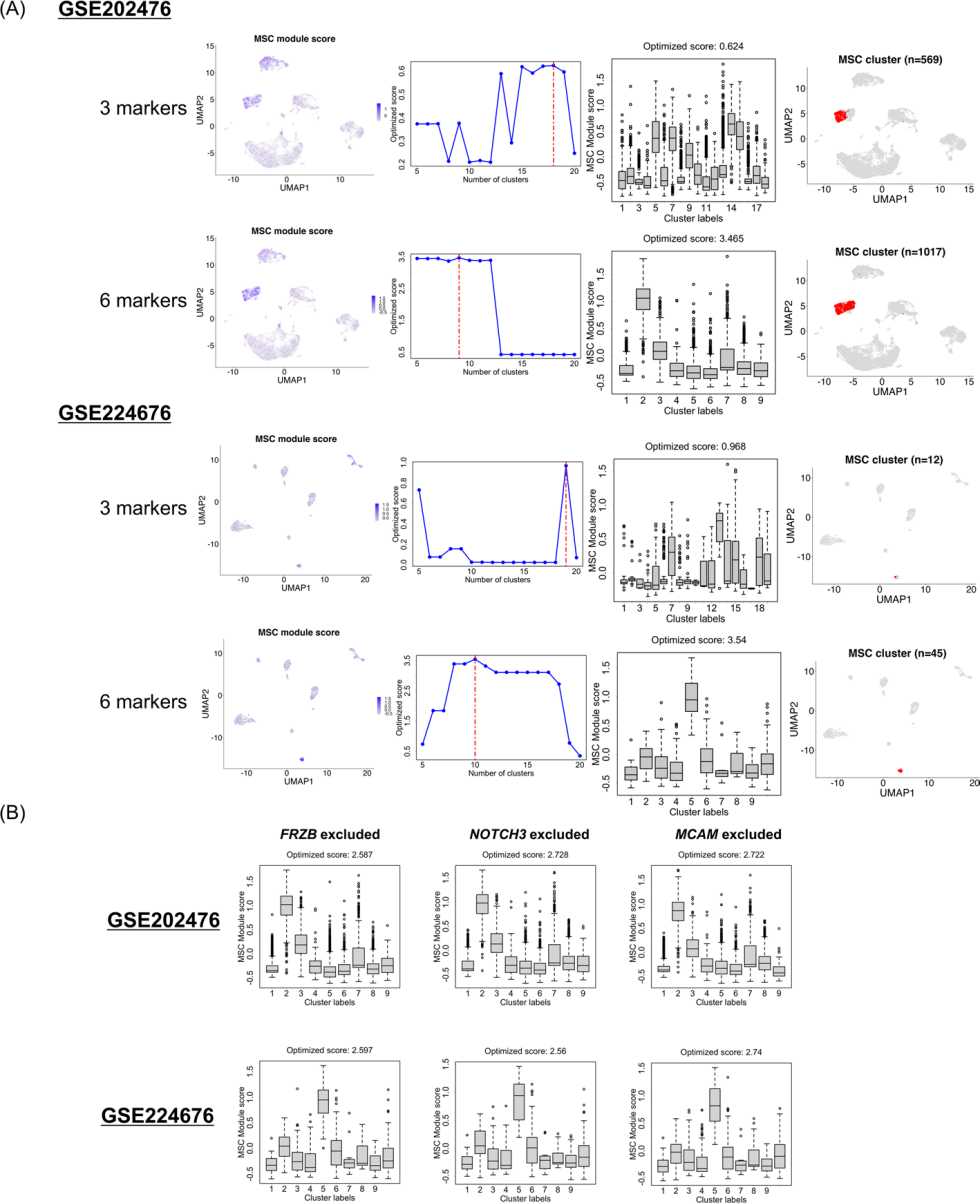
Examples of automated extraction of MSCs from human dental pulp scRNA-seq data using different marker sets. (**A**) Comparison of the three-marker set (*ENG, THY1, NT5E*) and the six-marker set (*ENG, THY1*, *NT5E, FRZB, NOTCH3*, and *MCAM*) using the proposed workflow. First column: module scores of the MSC marker sets projected onto UMAP. Second column: optimized score across different numbers of clusters. The selected number of clusters is indicated by a red dotted line. Third column: box-and-whisker plots of MSC marker-set module scores under the optimal clustering condition. Fourth column: MSCs identified under the optimal clustering condition. (**B**) Optimized scores using five-marker sets, excluding *FRZB, NOTCH3*, or *MCAM*

Using the six-marker set, the proposed workflow identified the MSC population with higher separation performance compared to the three-marker set (Fig. 2A). The first column of Fig. 2A shows the module scores projected onto UMAP, indicating the presence of a cell population with high scores. The second column in Fig. 2A presents the optimized scores for different numbers of clusters. Under optimal clustering conditions, the module score boxplots (third column) indicated a single cluster with a markedly elevated MSC module score that was clearly separated from the second-ranked cluster. UMAP visualization (fourth column) confirmed that this cluster formed a coherent population. Given the variability in RNA-level expression of cell surface markers, these three canonical genes alone were insufficient for robust identification of MSCs in dental pulp scRNA-seq data. In contrast, the six-gene marker set improved cluster separability and enabled stable extraction of a single MSC population.

The expression of representative negative control markers was examined (Supplementary Fig. 2). The hematopoietic marker, *PTPRC* (CD45), and endothelial marker, *PECAM1* (CD31), were specifically evaluated. The distribution of marker expression across clusters is shown as boxplots in Supplementary Fig. 2. Although occasional low-level outlier expression was observed, which is common in scRNA-seq data at the RNA level, MSC clusters identified by the proposed workflow were consistently negative for these markers. This indicates that the extracted populations were not substantially contaminated by hematopoietic or endothelial cells and that the six-marker set enables stable and robust identification of MSC populations across datasets.

To assess the validity of the expanded six-marker set for MSC identification, we systematically evaluated its contribution to performance within the proposed framework. The analysis was repeated using five marker sets in which one of the additional markers (*FRZB*, *NOTCH3*, or *MCAM*) was removed. Although the selected number of clusters remained identical to that obtained with the six-marker set, the optimized scores were consistently lower across datasets (Fig. 2B). This result suggests that the six-marker module score captured the MSC cluster more specifically. By quantifying cluster separation, this framework permitted the comparison of marker set performance and supported the use of a proposed six-gene marker set for MSC identification.

We examined whether the performance of the proposed marker set could be improved by adding a marker. For example, *NGFR* (CD271), which has been widely used as an enrichment marker for mesenchymal stromal cells [33], was added to the six-gene marker set, and the same workflow was applied (Supplementary Fig. 3). In GSE202476, the inclusion of *NGFR* resulted in a slight increase in module scores, whereas the optimal number of clusters selected by the workflow remained unchanged. In contrast, in GSE224676, the inclusion of *NGFR* led to a decrease in module scores and altered the optimal number of clusters selected by the workflow. These changes did not provide a clearer identification of the MSC population than the original six-gene marker set. The addition of *NGFR* did not improve MSC identification in these datasets and the original six-gene marker set was provisionally sufficient for consistent MSC extraction from dental pulp scRNA-seq data.

In addition to the k-means–based approach, a graph-based clustering strategy using the Louvain algorithm was evaluated. In this case, instead of specifying the number of clusters, the resolution parameter was systematically determined. This alternative analysis was conducted to confirm that the results were not dependent on the choice of the clustering method. In both GSE202476 and GSE224676, similar annotation results were obtained compared to those derived using k-means clustering. However, the optimized scores were lower than those obtained using k-means clustering in both datasets (Supplementary Fig. 4). The k-means approach provided robust and favorable performance for isolating MSC populations. Importantly, the proposed framework is not tied to a specific clustering algorithm but instead optimizes clustering granularity based on a biologically motivated criterion.

### Inflammatory transcriptomic features of MSCs under pulpitis indicated using pseudobulk analysis

Pulpitis is a common inflammatory disease of the dental pulp. Bulk RNA-seq studies of pulpitis have reported the activation of immune and DNA damage responses, with the suppression of proliferation at the tissue level [34, 35]. In a recent scRNA-seq study, Wu et al. evaluated ferroptosis-related changes across multiple cell types in dental pulp [18]. However, MSC-focused molecular and transcriptomic analyses are limited. The specific molecular alterations occurring within the MSC population in dental pulp are unclear.

**Fig. 3:**
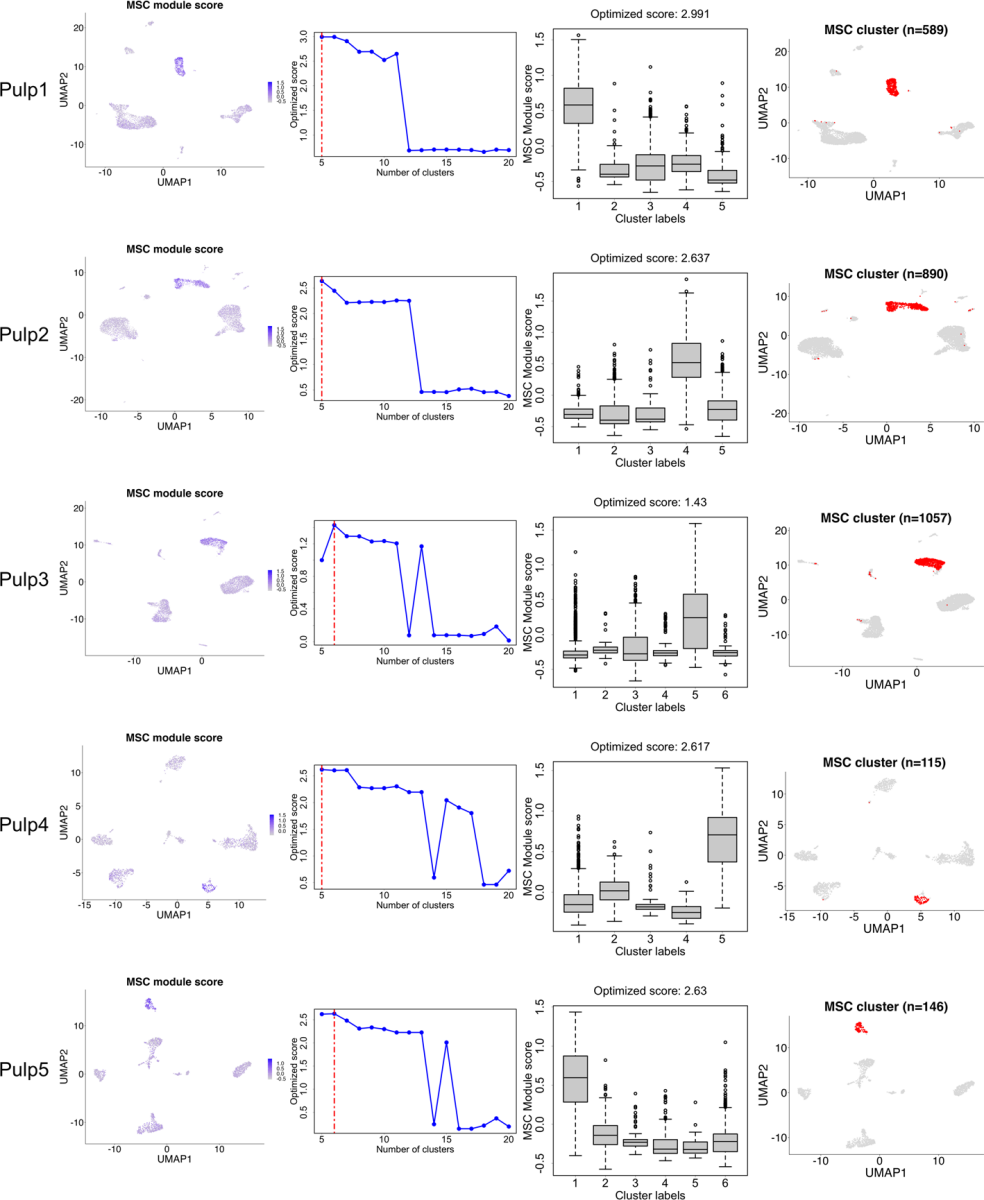
MSC extraction results in healthy donors’ dental pulp scRNA-seq data (GSE274562). Column descriptions are the same as in Fig. 2

**Fig. 4:**
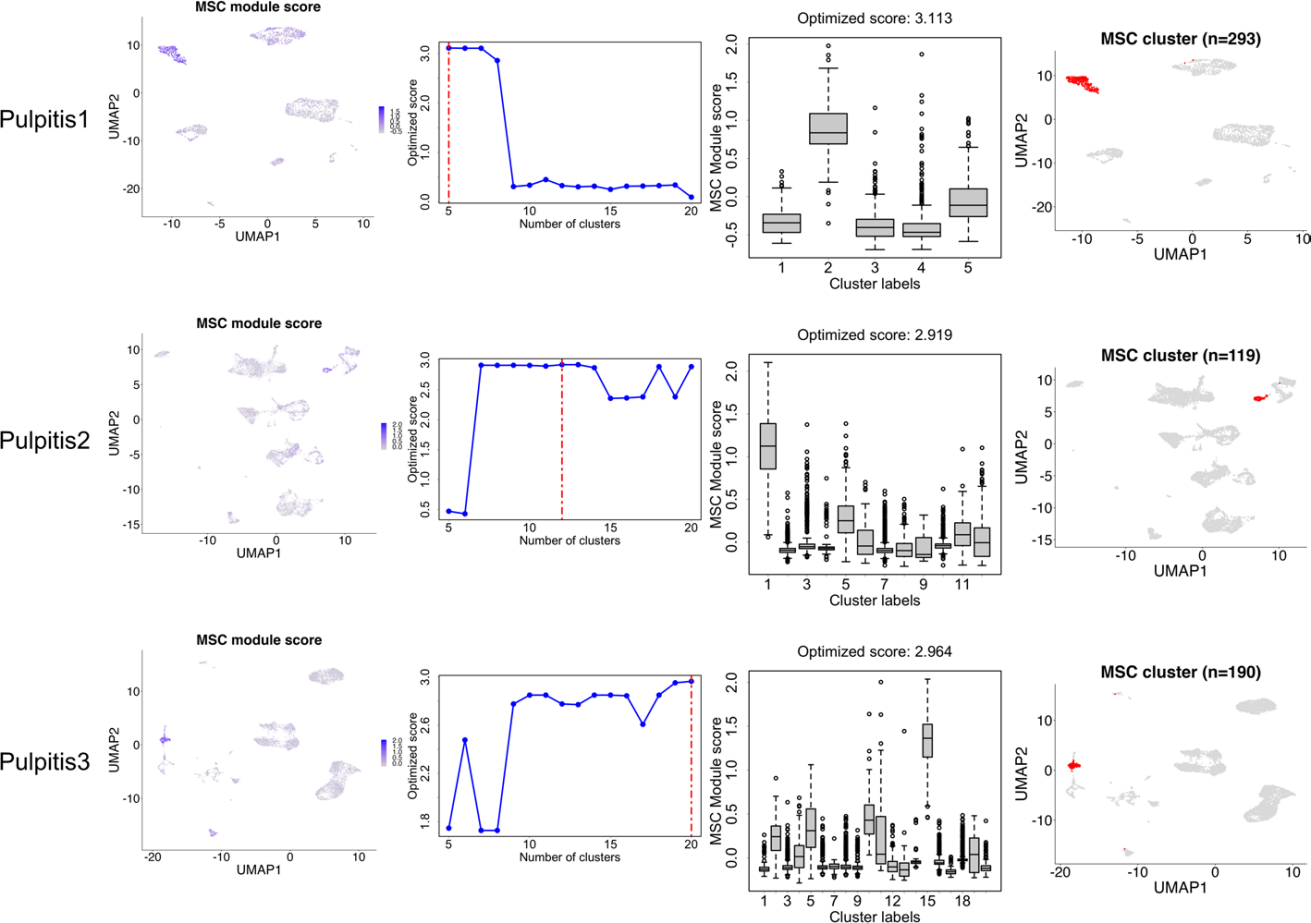
MSC extraction results from pulpitis donors’ dental pulp scRNA-seq data (GSE280528). Column descriptions are the same as in Fig. 2

Wu et al. generated the scRNA-seq datasets, GSE274562 and GSE280528, for healthy and pulpitis dental pulp samples [18], respectively. The current workflow was applied to all samples in these datasets to extract the MSC populations. Pseudo-bulk count matrices were constructed for the extracted MSCs, which yielded highly-enriched MSC populations across all samples. The results from the five healthy and three pulpitis samples are shown in Figs. 3 and 4, respectively.

**Fig. 5:**
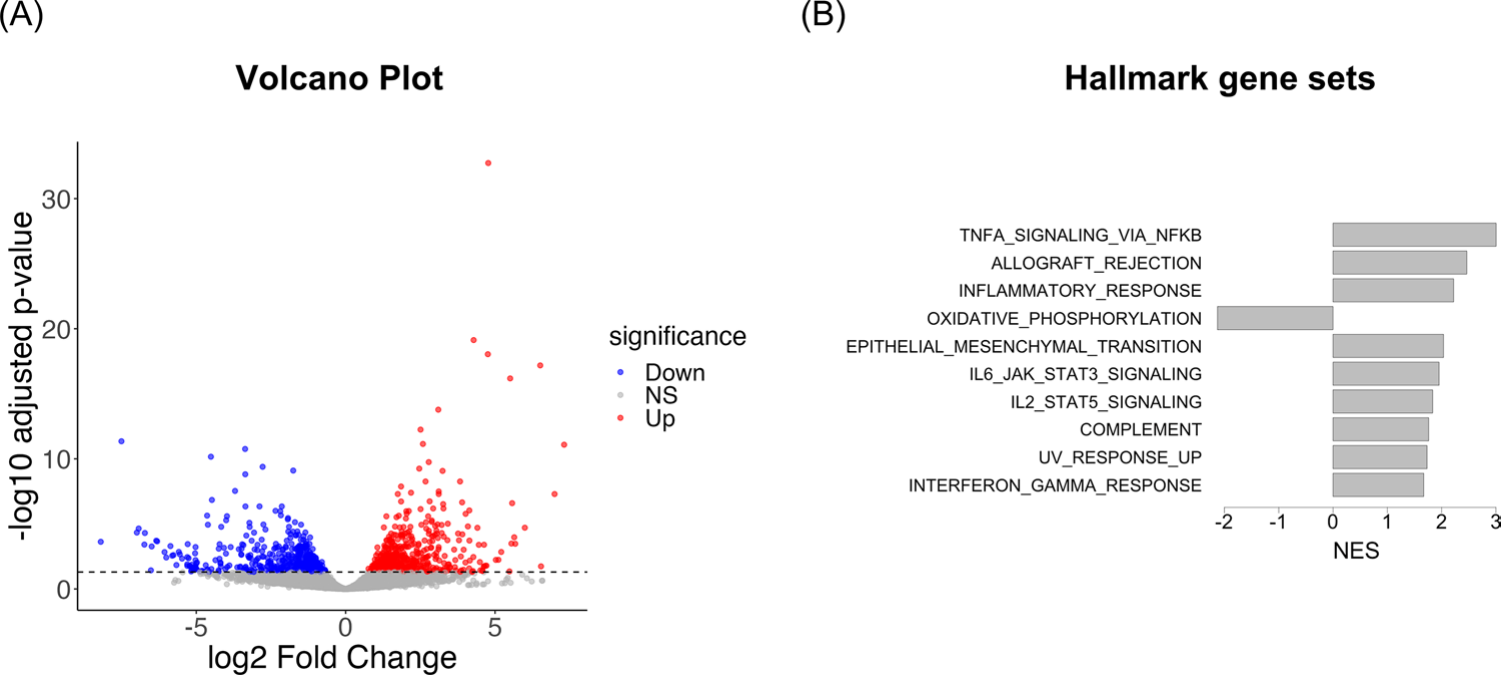
Pseudo-bulk transcriptomic comparison of MSCs in dental pulp between pulpitis and healthy donors. (**A**) Volcano plot illustrating differential gene expression in MSCs comparing donors with pulpitis and healthy donors. Genes significantly upregulated in pulpitis are shown in red, genes significantly downregulated in pulpitis are shown in blue, and non-significant genes (NS) are shown in gray. (**B**) GSEA based on log2 Fold change (pulpitis/healthy). Gene sets with adjusted *p*-value * < * 0.05 are shown, and the top 10 gene sets ranked by absolute normalized enrichment scores (NES) are displayed

Using pseudo-bulk count data, differential expression analysis was conducted to compare MSCs in control and pulpitis samples. In pulpitis samples, 564 genes were significantly upregulated and 576 genes were significantly downregulated (adjusted *P < 0.05)* (Fig. 5A). The complete DESeq2 results are presented in Supplementary Table 1. Gene set enrichment analysis (GSEA) based on $\textrm{log}_2$ fold changes showed that 21 of the 50 hallmark gene sets were strongly associated with transcriptome differences between pulpitis and healthy samples. Inflammation-related gene sets showed higher normalized enrichment scores (NES), indicating that MSCs adopted an inflammatory transcriptional phenotype under pulpitis conditions (Fig. 5B). The complete GSEA results are presented in Supplementary Table 2.

### Association between donor aging and the transcriptome of MSCs in dental pulp indicated using pseudobulk analysis

Age-related transcriptomic changes are generally associated with inflammatory signatures [36, 37]. However, bulk-omics data reflect mixtures of multiple cell types, and signals from broadly-distributed populations, such as immune cells and fibroblasts, can influence the results [38, 39]. These limitations highlight the importance of cell-type–specific analyses for evaluating intrinsic, age-related molecular changes. In a recent scRNA-seq study comparing young and aged dental pulp, Tong et al. reported age-related changes in cellular composition and identified *IGFBP7* as a marker of widespread upregulation across cell types [16]. However, their analysis emphasized SASP-related changes, and detailed evaluation of MSC-intrinsic molecular programs was limited.

Tong et al. conducted a two-group comparison between young and aged dental pulp samples. Their analysis was extended by incorporating two additional donors of known age (Fig. 2) and applying a Spearman correlation analysis, enabling a continuous assessment of the relationship between donor age and the pseudobulk transcriptome of MSCs in the dental pulp. Specifically, GSE274652, the dental pulp scRNA-seq dataset generated and analyzed by Tong et al. [16] was analyzed, which includes six donors—Control2 (18 year-old), Control1 (22 year-old), Control3 (26 year-old), Control6 (35 year-old), Control5 (45 year-old), and Control7 (>48 year-old)—and further integrated two previously-analyzed datasets, GSE202476 (13 year-old) and GSE224676 (27 year-old), resulting in a total of eight donors for the age association analysis.

**Fig. 6:**
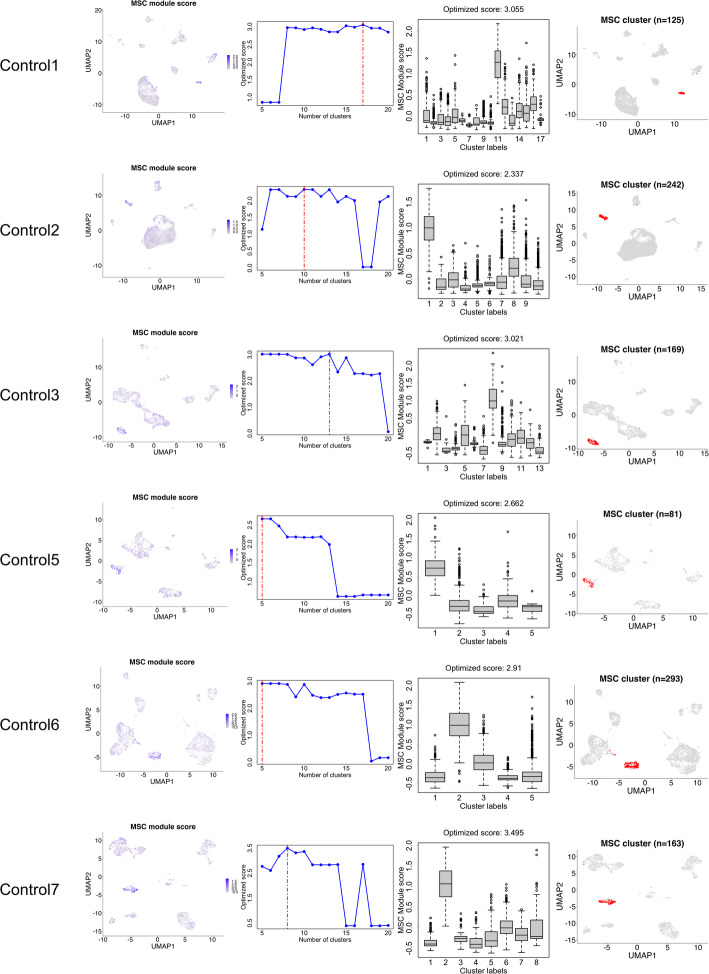
MSC extraction results in six donors’ dental pulp scRNA-seq data (GSE274652). Column descriptions are the same as in Fig. 2

MSCs were extracted using the proposed workflow (Fig. 6), and pseudo-bulk count data were generated for each donor. Common genes across datasets were retained, and counts per million (CPM) values were calculated. Gene expression levels were transformed into log(1 + CPM) values. Genes with mean expression values * > * 1 were used for downstream analysis.

**Fig. 7:**
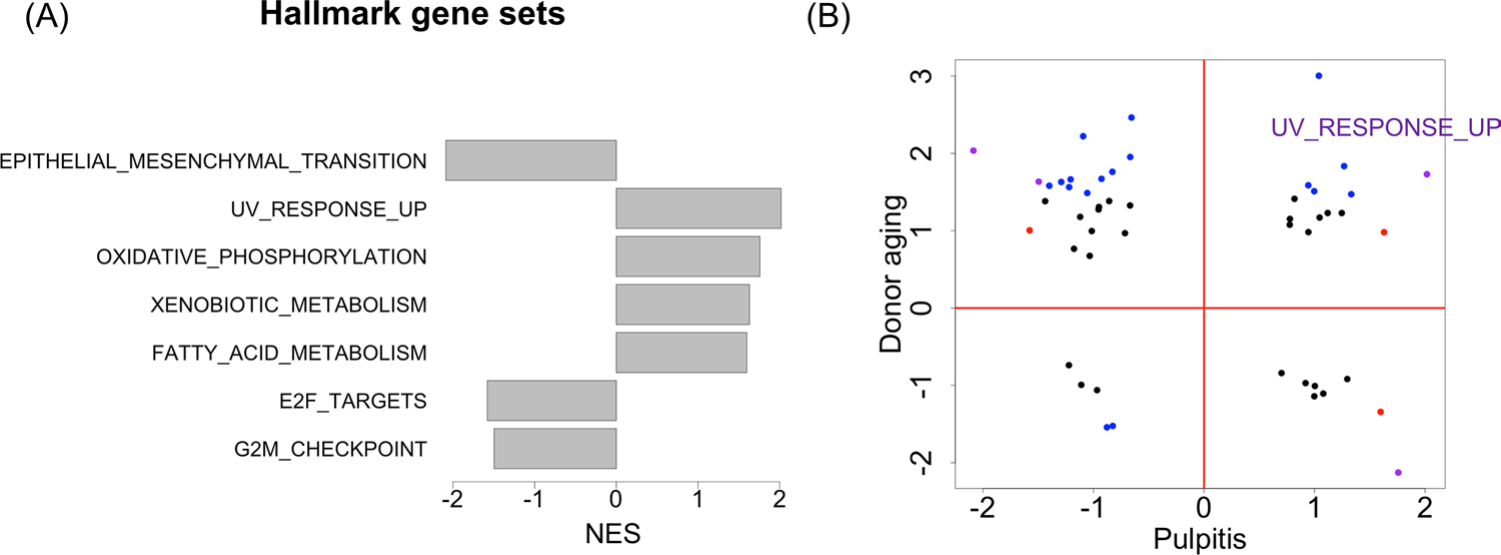
Pseudo-bulk transcriptomic association analysis of dental pulp MSCs with donor age (**A**) GSEA of Spearman correlation coefficients between gene expression value and donor age. Gene sets with adjusted *p*-value * < * 0.05 are shown. (**B**) Comparison of GSEA results between pulpitis and donor aging. Scatter plot showing the normalized enrichment scores (NES) of all 50 hallmark gene sets. Gene sets significantly enriched only in donor aging are shown in red, those significant only in pulpitis are shown in blue, and those significant in both conditions are shown in purple. Gene set UV_RESPONSE_UP was significantly associated in the same direction in both analyses

Spearman’s correlation coefficients were computed for donor age and gene expression levels. No genes reached statistical significance after Benjamini-Hochberg correction (adjusted *P < 0.05*), possibly because of the limited sample size (*n = 8*). GSEA was therefore conducted using Spearman correlation coefficients as the ranking metric. Six of the 50 gene sets showed strong associations (Fig. 7A). With increasing donor age, “EPITHELIAL_MESENCHYMAL_TRANSITION” was negatively enriched, suggesting attenuation of mesenchymal identity. In contrast, “UV_RESPONSE_UP,” “XENOBIOTIC_METABOLISM,” and “FATTY_ACID_METABOLISM” were positively enriched, indicating increased DNA damage–related responses and metabolic alterations. The complete GSEA results are presented in Supplementary Table 3.

The GSEA results for pulpitis and donor aging were compared across all 50 hallmark gene sets (Fig. 7B). In these gene sets, only UV_RESPONSE_UP was strongly associated in the same direction in both analyses, suggesting that both conditions enhanced stress-related responses in MSCs. In contrast, the inflammatory features observed in MSCs with pulpitis were not recapitulated in donor aging. For example, the gene sets TNFA_SIGNALING_VIA_NFKB and IL2_STAT5_SIGNALING, which showed high normalized enrichment score (NES) values in pulpitis, had NES values of 1.05 and 1.27, respectively, in the aging analysis. Although the direction of change was consistent, neither reached statistical significance. In contrast, IL6_JAK_STAT3_SIGNALING and INFLAMMATORY_RESPONSE exhibited NES values of *-0.66* and *-1.09*, respectively, in the aging analysis, indicating a trend in the opposite direction. These results suggest that, in dental pulp MSCs, the emergence of an inflammatory phenotype may not be a dominant feature of donor aging.

## Discussion

In this study, a framework for extracting MSCs from human dental pulp scRNA-seq data was developed. When analyzing dental pulp scRNA-seq data, MSCs are typically identified as clusters and annotated based on marker gene expression. In this process, the identification of MSCs depends not only on the choice of marker genes but also on clustering resolution. Coarse clustering may obscure MSC populations, whereas overly fine clustering may fragment them into multiple subclusters. To address this, we developed a framework that enables more robust and reproducible extraction of MSC populations by systematically optimizing clustering resolution based on a target cell-type module score.

A key characteristic of the proposed framework was that the clustering resolution is explicitly optimized for the extraction of dental-pulp derived MSCs. The method was designed to enhance the purity and separability of the MSC population by selecting the number of clusters that maximized the separation of MSC marker module scores. Consequently, the resulting clustering configuration was not intended to preserve the global structure of all cell types within the dataset. Therefore, this approach is not suitable for analyses aimed at comprehensively characterizing cellular heterogeneity in the dental pulp or whole-tooth scRNA-seq data. Instead, it should be regarded as a targeted strategy for isolating a specific cell population for downstream analyses, such as subclustering within MSCs or pseudo-bulk transcriptomic analysis. This design choice reflects a current gap in dental pulp research. Although whole-tissue profiling studies are increasing, systematic and reproducible extraction of MSC populations remains limited.

The selection of datasets in this study was based on the purpose of evaluating the proposed workflow under biologically interpretable conditions rather than exhaustively surveying all available dental scRNA-seq datasets. Although publicly available dental and dental pulp scRNA-seq datasets have increased in recent years, they remain heterogeneous in terms of donor number, sample source, disease status, developmental stage, sequencing platform, and analytical purpose. In particular, many datasets include only a single donor or a very small number of donors, which limits their utility for pseudo-bulk comparisons or for evaluating biological associations. Therefore, simply aggregating all available datasets would not necessarily provide a more rigorous assessment of the workflow and could instead reduce the interpretability of downstream analyses. In this study, we first used two independent dental pulp datasets as representative datasets to evaluate reproducible MSC extraction across studies, and then focused downstream analyses on datasets with multiple donors and the specific biological contrasts, namely pulpitis and donor aging. In the future, larger and more systematically designed dental pulp scRNA-seq datasets will allow this framework to be applied more comprehensively to evaluate inter-donor variability, disease-specific MSC states and age-related trajectories.

The proposed framework provides a systematic approach to evaluate marker sets for MSC identification by quantifying their ability to isolate a target population. However, this approach does not offer a fully systematic procedure for constructing marker sets. In the case of dental pulp, studies on MSC-specific markers are limited, and empirical exploration based on existing biological knowledge continues to play an important role in marker selection. In particular, many MSC-associated markers have been characterized at the cell surface protein level, and their correspondence to transcriptomic features is not always straightforward. For example, while STRO-1 is a classical MSC surface protein marker widely used for cell isolation [40], its corresponding gene or antigen is incompletely defined, making it difficult to incorporate directly into scRNA-seq–based analyses. In contrast, multimodal single-cell technologies, such as Cellular Indexing of Transcriptomes and Epitopes by Sequencing (CITE-seq), which simultaneously measure transcriptomes and cell surface protein expression [41], may enable a more systematic evaluation and refinement of marker sets by bridging RNA and protein-level information. The integration of these approaches could improve marker selection strategies and enhance the robustness of MSC identification in future studies.

A six-gene marker set consisting of *ENG*, *THY1*, *NT5E*, *FRZB*, *NOTCH3*, and *MCAM* enabled the consistent extraction of MSCs across multiple, independent, dental pulp datasets. Because cluster separability was explicitly quantified, this framework can be used to evaluate and refine marker gene sets. This provides a generalizable strategy for extracting specific cell populations in contexts where automated annotation is underdeveloped, such as MSCs from less-studied tissues like dental pulp, and facilitates cross-dataset pseudobulk analyses of purified target populations.

From a biological perspective, our results suggest that pulpitis and donor aging may be associated with different transcriptomic patterns in dental pulp MSCs. MSCs are widely recognized for their immunomodulatory and anti-inflammatory properties, but they can also acquire a pro-inflammatory phenotype and produce inflammatory cytokines under specific stimuli [42–44]. Consistent with this, MSCs from pulpitis samples showed enrichment of inflammation-related gene sets, supporting inflammatory transcriptional reprogramming in the setting of local pulp inflammation. In contrast, the aging analysis did not show similarly clear enrichment of inflammatory pathways, and instead suggested changes related to mesenchymal features, DNA damage response, and metabolism. However, this point should be interpreted with considerable caution. The donor age analysis was based on only eight samples, no individual genes reached statistical significance after multiple-testing correction, and subtle inflammation-related changes may therefore have been missed because of limited statistical power. These results suggest that, in dental pulp MSCs, the emergence of an inflammatory phenotype may not necessarily be a central feature of donor aging. However, further investigation will be needed to clarify whether donor aging induces inflammatory changes in this cell population.

## Conclusion

We propose an analytical framework that optimizes clustering resolution and enables extraction of MSC populations from dental pulp scRNA-seq data. By analyzing the extracted MSC pseudobulk data, transcriptomic changes in MSCs in the dental pulp associated with pulpitis and donor aging could be systematically evaluated. This framework is broadly applicable to the automated extraction of cell populations such as dental pulp–derived MSCs, for which an annotation method is not yet well established. This framework is expected to be widely useful in single-cell analyses.

## Electronic supplementary material

Below is the link to the electronic supplementary material.

Supplementary Material 1: Figures 1–4.

Supplementary Material 2: **Table 1**: Full DESeq2 results for mesenchymal stromal cells in the healthy vs. pulpitis.

Supplementary Material 3: **Table 2**: Full gene set enrichment analysis results for the healthy vs. pulpitis.

Supplementary Material 4: **Table 3**: Full gene set enrichment analysis results based on Spearman correlation coefficients between donor age and gene expression levels.

## Data Availability

The R code used in the study is available on GitHub at the following link (https://github.com/DaigoOkada/msc-extraction-dp).

## Data availability

No datasets were generated or analysed during the current study.
