# Supplementary material for: Automatic extraction of mesenchymal stromal cells from single-cell RNA-sequencing data of human dental pulp
Source: BioData Min. 2026 Jul 7;19:57. doi: 10.1186/s13040-026-00581-x (PMC13435457; doi:10.1186/s13040-026-00581-x)
Supplement: Supplementary file 1 [file 13040_2026_581_MOESM1_ESM.pdf]

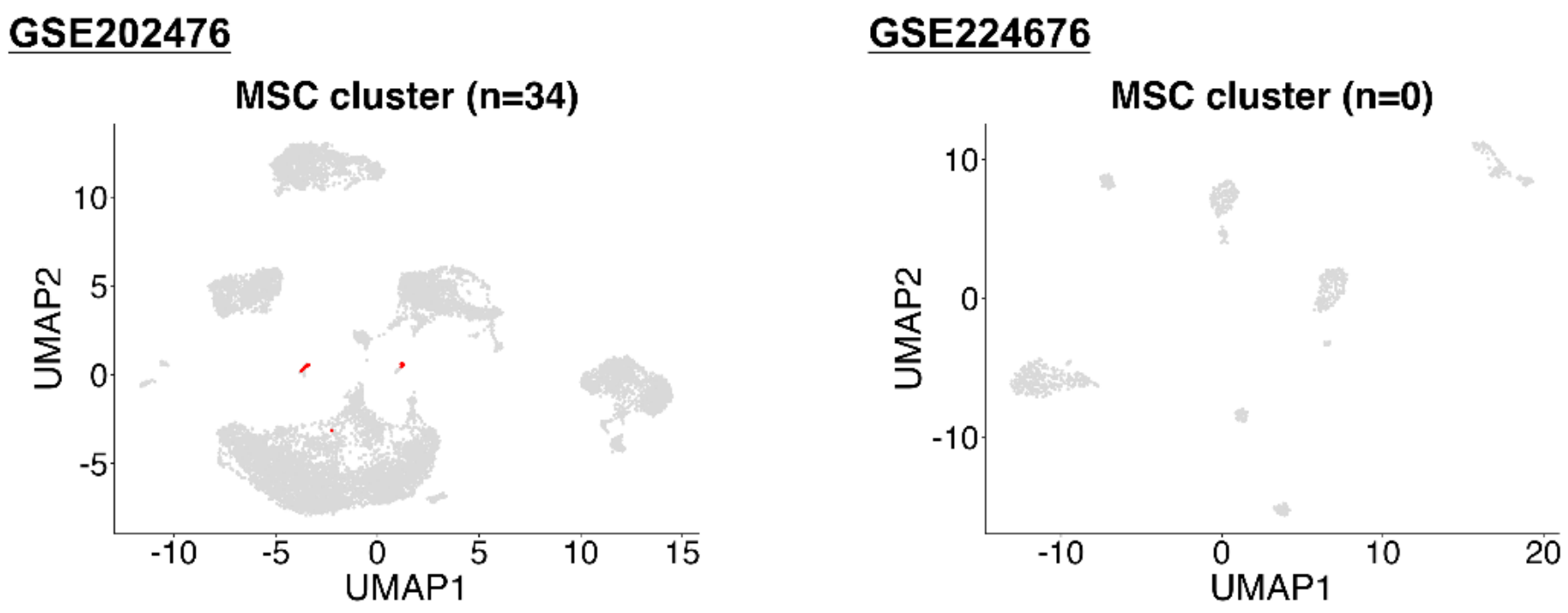

Supplementary Figure 1. Results of automated MSC annotation using SingleR in two human dental pulp scRNA-seq datasets. In GSE202476, the cells annotated by MSC were not assigned to a single coherent population, but were instead fragmented across multiple clusters. In GSE224676, no cells were not annotated as MSC. These results indicate that reference-based annotation may not reliably capture MSC populations in dental pulp scRNA-seq data.

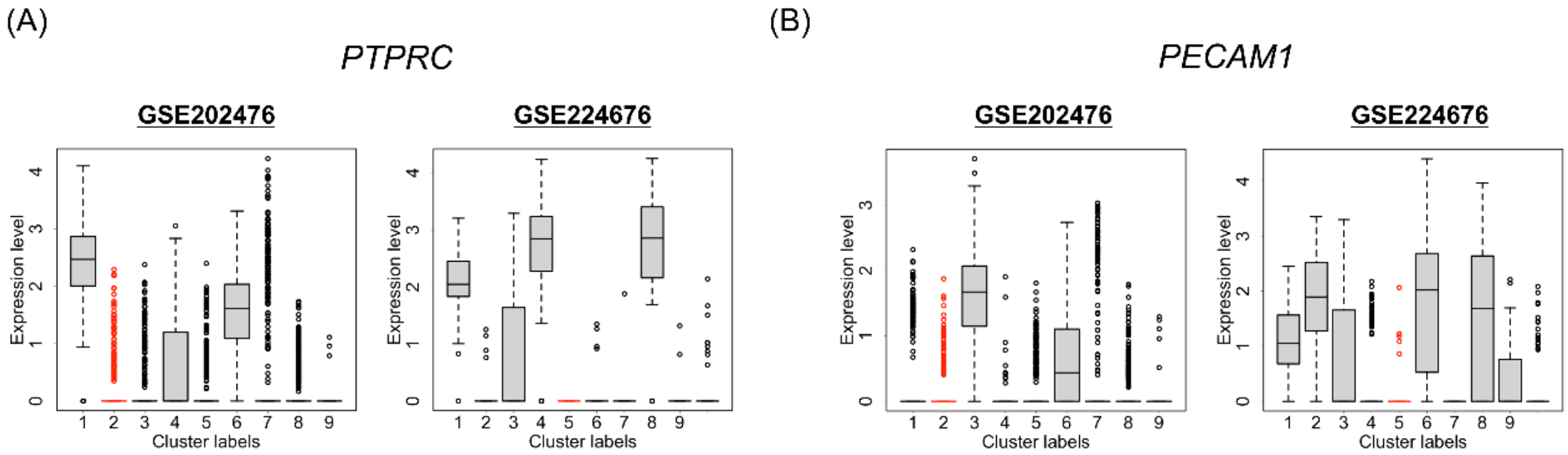

Supplementary Figure 2. Box-and-whisker plots showing the expression levels of the MSC negative markers *PTPRC* and *PECAM1* across clusters. The cluster identified as MSC is highlighted in red.

GSE202476

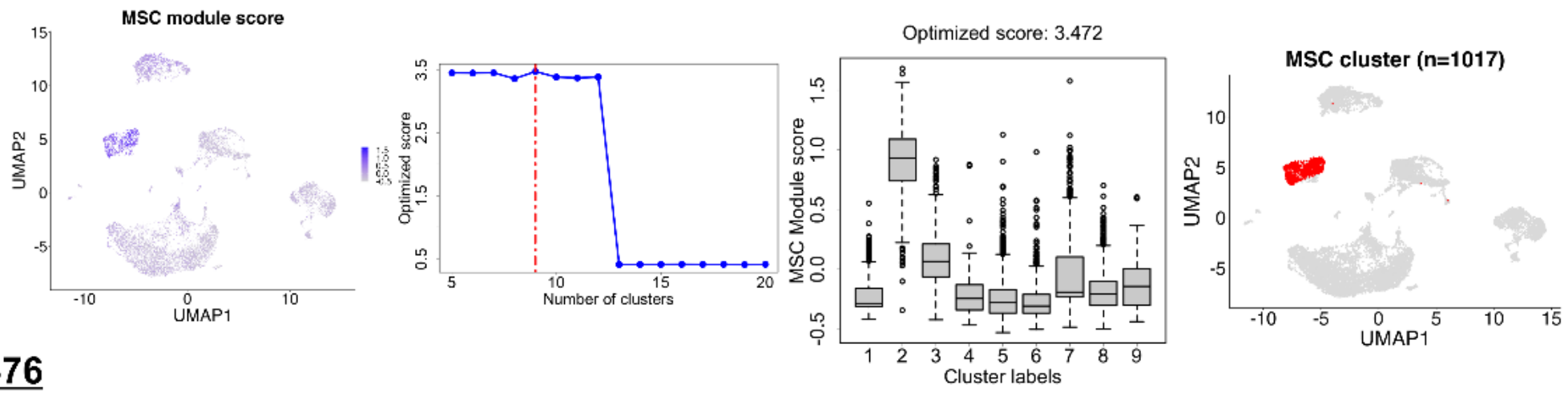

GSE224676

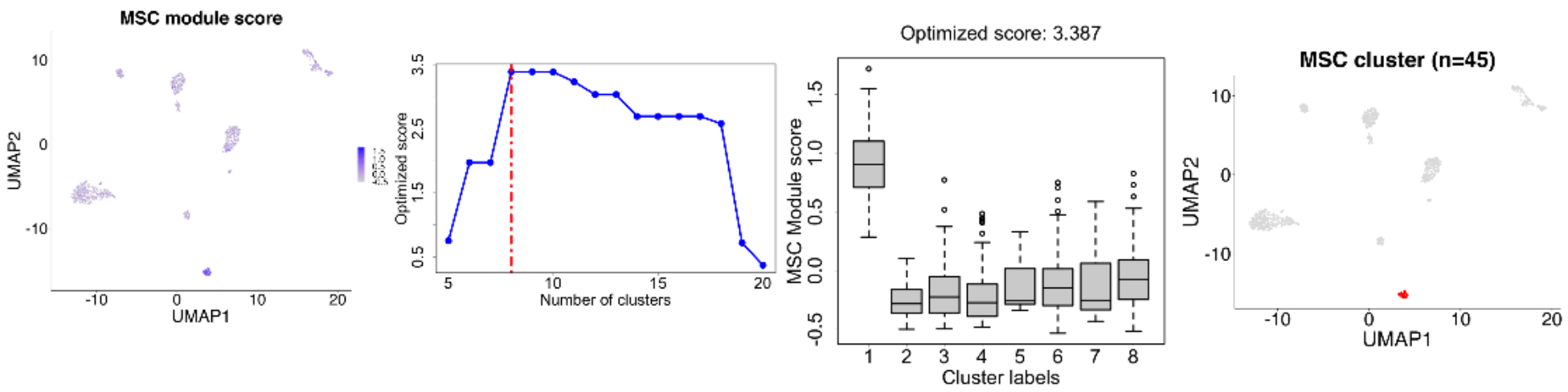

Supplementary Figure 3. Results of applying the proposed workflow using a seven-marker set, in which *NGFR* was added to the proposed six-marker panel.

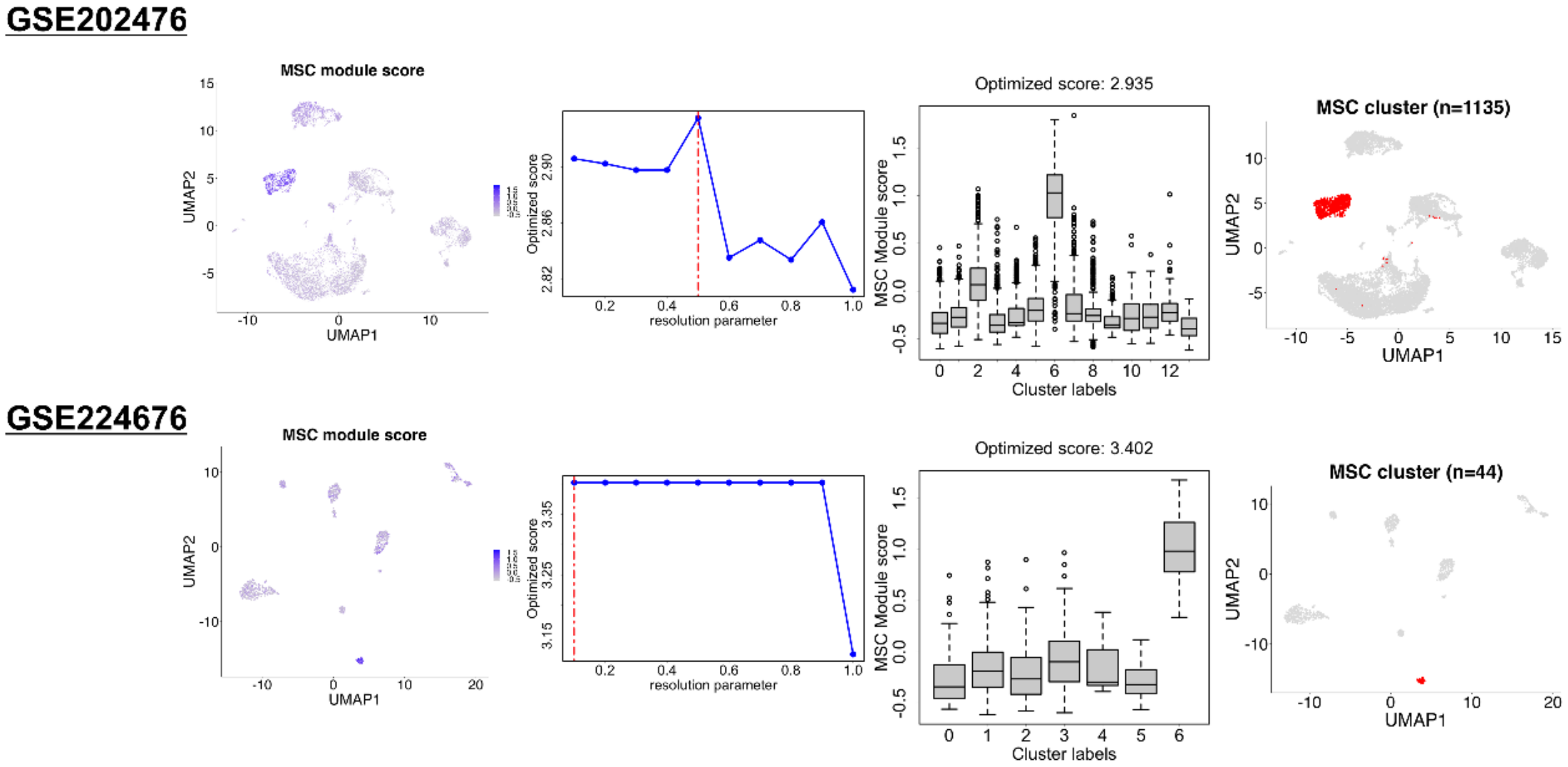

Supplementary Figure 4. Results of applying the workflow using Louvain clustering. The resolution parameter in Seurat's *FindClusters* function was systematically optimized.
